# Supplementary material for: Multi-level patterning nucleic acid photolithography
Source: Nat Commun. 2019 Aug 23;10:3805. doi: 10.1038/s41467-019-11670-3 (PMC6707258; doi:10.1038/s41467-019-11670-3)
Supplement: Supplementary file 1 — Supplementary Information [file 41467_2019_11670_MOESM1_ESM.pdf]

Supplementary Material

Multi-Level Patterning Nucleic Acid Photolithography

Kathrin Hölz, Erika Schaudy, Jory Lietard* and Mark M. Somoza*

*Correspondence: jory.lietard@univie.ac.at or mark.somoza@univie.ac.at

Institute of Inorganic Chemistry, Faculty of Chemistry, University of Vienna, Althanstrasse 14 (UZA II), 1090 Vienna, Austria

Supplementary Methods

Substrate preparation. Schott Glass D microscope slides were functionalized with N-(3-triethoxysilylpropyl)-4-hydroxybutyramide (Gelest SIT8189.5). The slides were placed in a stainless steel rack and gently agitated in a solution of 2 % (v/v) of the silane and 0.1 % acetic acid in 95:5 ethanol/water. After 4 hours at room temperature, the slides were rinsed twice for 20 min in the 95:5 aqueous ethanol and allowed to cure for a minimum of 2 hours at 120 °C under vacuum. After cooling overnight to room temperature under vacuum, the slides can be stored in a desiccator until use.

Maskless synthesis. The Maskless Array Synthesizer (MAS) instrument can be conceptually divided into two components, an optical system and a chemical delivery system. The chemical side consists of an Expedite 8909 nucleic acid synthesizer, which is used to pump solvents and reagents to the functionalized glass surface where biochip synthesis takes place. The optical system is similar to that of an I-line photolithography system, but it uses a digital micromirror device (Texas Instruments 0.7 XGA DMD) with 1024 × 768 individually addressable mirrors in place of photomasks to pattern the ultraviolet light from a mercury lamp, or ultraviolet LED.

Light from a 365 nm high-power UV-LED (Nichia NVSU333A) is focused into a reflective homogenizing light pipe with a rectangular cross section of 6 × 8 mm. Spatially homogenized light exiting the homogenizer is magnified by a factor of two and imaged onto the DMD. The DMD itself is imaged onto the synthesis surface with an Offner relay. The Offner relay consists of two spherical mirrors, a primary mirror with a concave radius of curvature of $450.00^{+0.0}_{-0.3}$ mm of diameter 200 mm, and a convex secondary mirror with 225.29±0.1 mm radius of curvature and a diameter of 36.6 mm. The radiant intensity of light reaching the reaction cell image plane was measured using a calibrated SÜSS Model 1000 UV intensity meter with a 365 nm probe (SÜSS MicroTec). The typical intensity used for these experiments was ~80 mW/cm^2.

The pattern displayed on the DMD is imaged onto the synthesis surface, where layout and sequences are determined by selective removal of the photocleavable protecting groups. Reagent delivery and light exposures are synchronized and controlled by a computer, which also stores and orders the display of virtual masks on the DMD. The chemistry is similar to that used in conventional solid-phase synthesis. The primary modification is the use of phosphoramidites with a photocleavable protecting group. Upon absorption of a photon near 365 nm, and in the presence of a weak organic base, the Bz-NPPOC (DNA) or NPPOC (all other monomers) groups drop off, leaving a hydroxyl terminus which reacts with an activated phosphoramidite during the next synthesis cycle.

Phosphoramidites and chemistry. The DNA phosphoramidites were obtained from Orgentis while the non-DNA phosphoramidites were obtained from ChemGenes. The phosphoramidites were activated using 0.25 M 4,5-dicyanoimidazole in acetonitrile (Biosolve Chimie). The exposure solvent consisted of 1 % (m/v) imidazole (Sigma-Aldrich 56750) in anhydrous DMSO (Biosolve Chimie). The oxidizer was tetrahydrofuran/water/pyridine/iodine 90.54/9.05/0.41/0.43 (v/v/v/w) (Sigma-Aldrich L860021). The synthesis washing steps used anhydrous acetonitrile (Biosolve Chimie). High purity helium was used as blanket gas over the synthesis solvents and reagents, and also in the drying step of each cycle.

Synthesis efficiency and yield. Maskless photolithographic nucleic acid synthesis makes use of lightly modified standard phosphoramidites chemistry. As a result, the chemical synthetic yields are comparable, with stepwise coupling yields of about 99 % to 99.9 % for DNA and DNA-like monomers (reverse synthesis monomers, dU, 2′-deoxy-2′-fluoro-β-d-arabinonucleic acid (FANA), dNaM, etc.), ~99 % for RNA monomers. The coupling efficiency of other monomer phosphoramidites, including branches and fluorophores are highly variable, but can be considered similar to those obtained in standard solid phase synthesis. Nevertheless, in our experience, coupling times in maskless photolithographic nucleic acid synthesis can be reduced by up to a factor of 4 relative to standard coupling, presumably due to the improved accessibility on the smooth planar substrate and much larger excess of phosphoramidites relative to surface reactive sites. Overall error rates are higher due to imperfect optical contrast, primarily due to diffraction and scattering of light from mirror edges and imperfections on the optical surfaces. The additional error rate depends strongly on the spacing between independently addressed surface features (“spots”). The length of chip-synthesized oligonucleotides is potentially much longer than can be achieved in standard solid-phase synthesis because the surface is never exposed to the strongly acidic conditions that can lead to depurination and strand cleavage. In the experiments presented here we synthesized up to 85mers and have synthesized up to 150mers in other experimental contexts. Oligonucleotides of these lengths hybridize well and specifically, but we do not yet know the precise error rates.

Deprotection for biochips with no RNA. After the synthesis, the nucleobase and phosphodiester protecting groups are removed by immersing the biochips in 1:1 (v/v) ethylenediamine/ethanol (EDA/EtOH) for two hours at room temperature, then washed twice in deionized water, dried with argon and stored in a desiccator.

Deprotection for biochips with RNA. Deprotection of RNA monomers additionally requires removal of the ALE 2′-OH protecting group, which must happen after removal of the cyanoethyl protecting group. In order to do this, the slides were deprotected in a 2:3 solution of anhydrous triethylamine in acetonitrile (ACN) for 1h30 at r.t. (50 ml in a Falcon

tube) with gentle agitation. The biochips were then rinsed twice in ACN (20 ml), dried in a microcentrifuge, then transferred into a 0.5 M solution of hydrazine hydrate (1.2 ml) in pyridine/acetic acid 3:2 (50 ml in a Falcon tube) for 2 h at r.t. The biochips were washed twice in ACN (20 ml each), then dried in a microcentrifuge. Finally, for biochips containing both DNA and RNA nucleotides, a final deprotection step consisted in shaking in a 1:1 solution of EDA in EtOH for 1 h at r.t. The resulting biochips were washed with nuclease-free water (2 × 20 ml), dried then stored in a desiccator until further use.

Hybridization and fluorescent image capture. The biochips were hybridized to Cy3-, Cy5 and FAM-labeled complementary DNA oligonucleotides purchased from Eurogentec, as described by Sack et al. Oligonucleotide sequences together with their modifications are listed in Supplementary Table 1.

Supplementary Table 1. Sequences of complementary oligonucleotide probes used in Figure 2.

#	Probe name	Color level	Sequence (5' to 3')	Modification	Chemistry
**1**	GLD1-Cy3_DNA	G0	T	5'-Cy3	DNA
		G36	TTA CCA TAG AAT CAT GTG CC		
		G72	TTA CCA TAG AAT CAT GTG CCA TAC		
		G109	TTA CCA TAG AAT CAT GTG CCA TAC ATC		
		G145	TTA CCA TAG AAT CAT GTG CCA TAC ATC		
		G182	TTA CCA TAG AAT CAT GTG CCA TAC ATC A		
		G218	TTA CCA TAG AAT CAT GTG CCA TAC ATC AT		
		G255	TTA CCA TAG AAT CAT GTG CCA TAC ATC ATC		
**2**	Click-Cy5_DNA	R0	G	5'-Cy5	DNA
		R36	GAA GCA GAT GGA ATC GA		
		R72	GAA GCA GAT GGA ATC GAT TC		
		R109	GAA GCA GAT GGA ATC GAT TCG		
		R145	GAA GCA GAT GGA ATC GAT TCG G		
		R182	GAA GCA GAT GGA ATC GAT TCG GT		
		R218	GAA GCA GAT GGA ATC GAT TCG GTG		
		R255	GAA GCA GAT GGA ATC GAT TCG GTG A		
**3**	QC25-FAM_DNA	B0	G	5'-FAM	DNA
		B36	GTC ATC ATC ATG AAC CAC		
		B72	GTC ATC ATC ATG AAC CAC C		
		B109	GTC ATC ATC ATG AAC CAC CCT		
		B145	GTC ATC ATC ATG AAC CAC CCT G		
		B182	GTC ATC ATC ATG AAC CAC CCT GG		
		B218	GTC ATC ATC ATG AAC CAC CCT GGT		
		B255	GTC ATC ATC ATG AAC CAC CCT GGT C		

The deprotected biochips were hybridized in a self-adhesive hybridization chamber (Grace Biolabs SA200) with 300 µl of a hybridization mix consisting of 150 µL 2x MES hybridization buffer (100 mM MES, 1 M Na^+, 20 mM EDTA, 0.01 % Tween20), 110 µL nuclease free water, 13.3 µL acetylated BSA (10 mg/ml) and 26.7 µL of the 100 nM labeled complementary oligonucleotide. After incubation for 2 hours in a hybridization oven (Boekel Scientific) at 42 °C, the biochips were washed in non-stringent wash buffer NSWB (SSPE; 0.9 M NaCl, 0.06 M phosphate, 6 mM EDTA, 0.01 % Tween20) for 2 minutes, in stringent wash buffer SWB (100 mM MES, 0.1 M NaCl, 0.01 % Tween20) for 1 minute, followed by a short wash in final wash buffer FWB (0.1x SSC) for a few seconds, after which they were dried in a microarray centrifuge. Scanning was performed at 2.5 µm resolution using a GenePix 4400A equipped with lasers and filters for red, green and blue imaging.

Conversion of images to virtual masks. In Photoshop, we converted the images to RGB and reduced their pixel size to the dimensions of the DMD or lower. Here we used a DMD with dimensions of 1024 x 768 but made images with dimensions 1018 x 762 to preserve space at the margins for hybridizable fiducial features for easy overlay of the fluorescent images. Smaller images shared the available area with replicates or other images.

Photoshop was also used to reduce the number of gray levels in each color channel to the desired number. This can be done using the “posterize” menu item and specifying the number of grays (e.g. 8). Once this is done for all three colors, the channel tab is chosen and a single channel is made visible. Then, this image is copied into a new file. To convert this to spreadsheet format, we use a simple Matlab program "grayimagetoarray.m" consisting of the following lines of code:

```
[file,path] = uigetfile('C:\*.tif');
if isequal(file,0)
  disp('User selected Cancel');
else
  disp(['User selected ', fullfile(path,file)]);
end
I = imread(fullfile(path,file));
filename = strcat(path,'image.xlsx');
xlswrite(filename,I)
```

This generates a spreadsheet with the same dimensions as the original image, but with a number between 0 and 225 in each cell indicating the grayscale value (0, 36, 72, 109, 145 182, 218, 255 in the case of the 3 bit grayscales used here; Table S1). More complex grayscales require longer oligonucleotides and/or more complex modifications, such as mismatches, to achieve the required level of melting temperature tuning. Each of these numerical values is replaced with a sequence calibrated to result in the appropriate intensity upon hybridization (Table S1). This process generates

three spreadsheets (R,G,B) which are concatenated to generate a spreadsheet with all necessary sequences in the design and their desired locations on the biochip.

The spreadsheet is converted to a single column text list with 775,716 rows. This serves as the input for our chip design program, which generates the virtual masks (one-bit bitmap images) that are sent sequentially to the DMD to tilt mirrors to either the on or off positions.

Palette and color rendering accuracy. The fluorescent color palette generated by the method described above is a reasonable approximation to the colors of the original images, but as can be seen in Figure 2, the colors are not exact matches. Supplementary Figure 1 shows more details of the color differences between the original images and their reproduction in DNA. The color discrepancy is due to a number of factors: (1) inexact color matching in each of the three fundamental RGB color channels, (2) fluorescent energy transfer (FRET) between fluorescent DNA labeling dyes, (3) hybridization variability, (4) readout channel crosstalk, and (5) color overlay error. (1) Inexact color matching refers to the attribution of a color level (e.g. G109) with a specific surface oligonucleotide (e.g. TTA CCA TAG AAT CAT GTG CCA TAC ATC). These matches (Supplementary Table 1) are based on calibrations carried out on all possible truncations to the three sequences. The desired color space of 3 bits results in the need for 8 sequences per channel which in turn, result in equally spaced hybridization intensities between 0 and 255 of the original 8 bit color depth. The available truncations do not result in exact intensity matches. This could be improved by generating intensity calibrations based also on single or multiple mismatches. The resulting finer gradations would allow better matches between DNA sequences and the desired intensity level, as well as deeper color spaces. Nevertheless, other sources of color error likely limit the maximum color accuracy that can be obtained. (2) FRET refers to non-radiative energy transfer between labeling dyes resulting from proximity and overlap in their emission and absorption spectra. Such effects are relatively small in comparison to overall fluorescence intensity in this experimental context, but are sufficient to cause a noticeable color shift. There is spectral overlap between fluorescein and Cy3 and between Cy3 and Cy5. This can be mitigated by increasing the spacing between dyes by the addition of spacer sequences and/or through more complex intensity calibrations that account for FRET. (3) Hybridization variability is due to inconsistent levels of hybridization due to variations in oligonucleotide concentration, hybridization temperature and salt concentrations, as well as differences in handling of the surface during hybridization, washing and drying. Hybridization, washing and drying steps are relatively complex manual operations that are very difficult to automate and can result in significant variability across time. In addition, hybridization intensity gradients across the surface can often be observed that can be attributed to the flow patterns of the hybridization or wash solutions. Hybridization

error can be mitigated by accurate handling and robust hybridization protocols. (4) Readout channel crosstalk is due to partial overlap in the absorption spectra of the fluorescent dyes as well as bandpass limitations of the colored filters used in the collection optics. For example, the 488 nm laser used to excite fluorescein (blue channel) partially overlaps the tail of the Cy3 absorption spectrum (red channel).

On the emissions side, partial overlap of emission spectra means that these are difficult to completely separate. This effect can be seen in Supplementary Figure 7, where partial excitation of Cy3 fluorescence by the blue (488 nm) laser results in a weak image in the green channel. These effects are potentially addressable through the optimization of excitation wavelengths and by using filters in the fluorescence collection optics with reduced bandpass to better exclude light originating from other chromophores. Also on the readout side of the experiment, is color overlay error (5). In our system, surface fluorescence is read by a rastering optical system that scans a laser over the surface and records the resulting fluorescence emission. The three colors are read in three consecutive scans. Due to both mechanical factors and color dependent optical path factors, the three scans do not superimpose exactly. The misalignment is small, on the order of the readout resolution of 2.5 microns, but this is enough to result in color distortion at the interstices of synthesis pixels. This particular color error could be improved with a higher resolution fluorescence scanner or a scanner with a sensor (such as a charge coupled device) that captures the entire image at once.

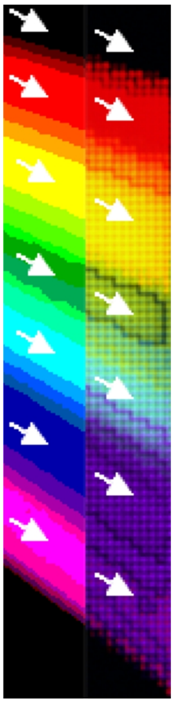

Supplementary Figure 1. Original RGB color (DNA color reproduction). Detail from prism image in Fig. 2.

Black: 0,0,0 (0,0,3)

Red: 255,0,0 (229,1,2)

Yellow: 255,255,0 (240,236,3)

Green: 0,170,0 (121,148,19)

Light blue: 0,255,255 (116,241,216)

Dark blue: 0,0,170 (94,0,164)

Purple 255,0,255 (111,1,174)

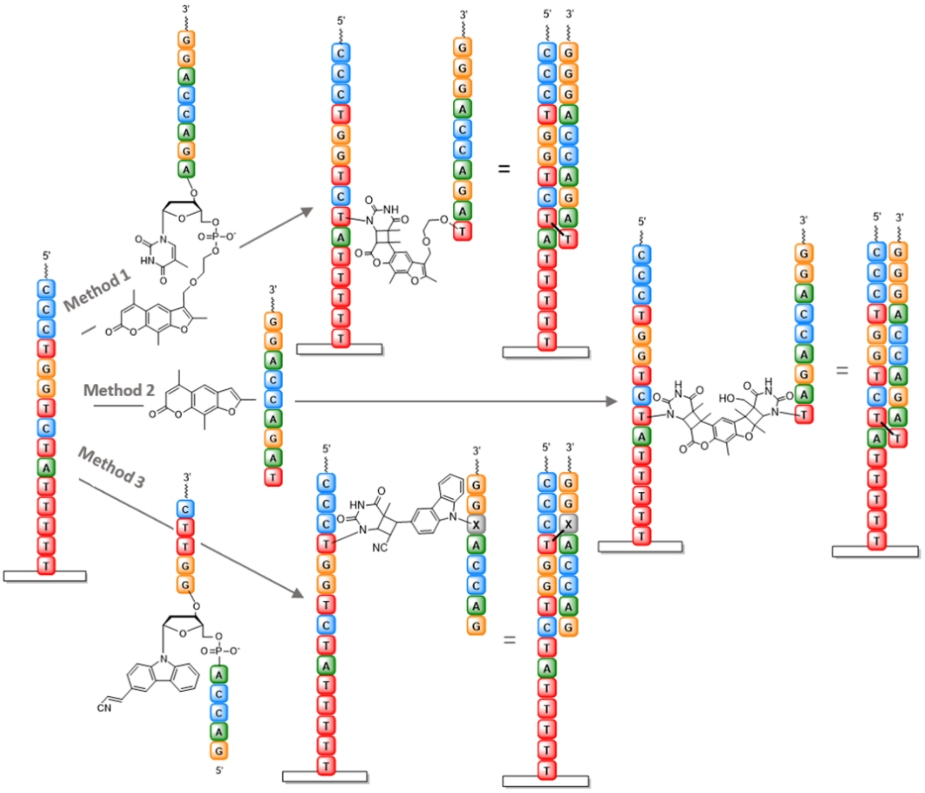

Supplementary Figure 2. Scheme for the photo-induced crosslinking of oligonucleotides on biochips using either oligonucleotides 5'-modified with 4,5',8-trimethylpsoralen *via* ethylene spacer (tethered psoralen; Method 1), free 4,5',8-trimethylpsoralen (free psoralen; Method 2) or 3-cyanovinylcarbazole-modified (^{CNV}K) oligonucleotides (Method 3).

Crosslinking efficiency. A scheme of the strategies for the photo-induced crosslinking of oligonucleotides on biochips is shown in Supplementary Figure 2. In order to determine the efficiency and final yield of the different photo-induced crosslinking methods on biochips and to establish the optimal settings for future use, photo-crosslinking experiments were performed using a gradient of UV light exposure. Using maskless array *in situ* synthesis (MAS), arrays of single-stranded 32mer DNA sequences were synthesized. After deprotection, the DNA sequences were hybridized to their complementary strands, either carrying a tethered 4,5',8-trimethylpsoralen (TMP, or psoralen) at the 5'-end (method 1) or a ^{CNV}K unit (method 3), or in presence of free psoralen (method 2), depending on the method under investigation. The probes were then exposed to 365 nm UV light at a radiant intensity of 80 mW/cm^2 from a high-power UV LED. The gradients of exposure on the surface were created using mirror patterns on the DMD so as to allow for the exposure of defined subsections of the surface for different time periods. To prevent unintended UV exposure due to light scattering, the exposed areas were separated by unexposed gaps. After photo-crosslinking, the surfaces were washed thoroughly in MilliQ water at room temperature for 6 minutes on an orbital shaker (25 rpm) to completely remove any non-crosslinked oligonucleotides. The following washing steps in non-stringent and stringent

hybridization buffers allow for a rebuffering of the nucleic acid strands as well as the attached Cy3-labels, promoting duplex formation and providing the counterion for optimal dye fluorescence. A short final washing step with 0.1x SSC buffer removed residual salt from the surface before scanning. The chosen unmodified DNA sequences all had a sufficiently high melting temperature (>56 °C) to ensure strong hybridization. Additionally, some psoralen derivatives are known to exhibit binding based on non-covalent interactions, which further increases the effective hybridization temperature. The washing procedure subsequent to the UV light exposure is therefore a crucial step to distinguish crosslinking from stable hybridization. Appropriate washing protocols were carefully developed and evaluated in preliminary studies. Radiant UV light exposure gradients were recorded for all three different crosslinking methods and for different oligonucleotide chemistries. The crosslinking yield as a function of UV exposure is shown in Supplementary Figure 3 (duplicate of Fig 3c).

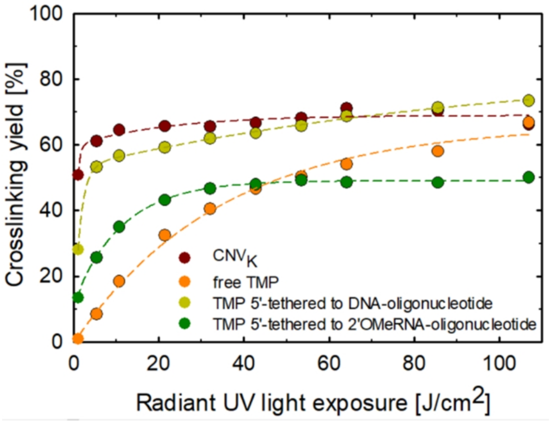

Supplementary Figure 3. (Duplicate of Fig. 3c) Radiant UV light exposure gradients for different crosslinking methods. A high-power UV LED served as the light source, providing UV light at a wavelength of 365 nm and at an intensity of 80 mW/cm^2. Crosslinking was performed using either ^{CNV}K-modified oligonucleotides (*dark red*), free psoralen (*orange*), psoralen-modified DNA oligonucleotides (*light green*) or psoralen-modified 2'-*O*Me-RNA oligonucleotides (*dark green*). All data points for any given crosslinking method were recorded on a single surface.

As expected, due to their high photo-reactivity, oligonucleotides carrying a ^{CNV}K modification showed the fastest crosslinking rate, plateauing at around 70 % yield. A relatively similar curve profile, albeit with slightly reduced crosslinking efficiency at lower radiant exposures, could be observed for the 5'-psoralen modified oligonucleotides. However, rather than plateauing, the crosslinking yield with 5'-psoralen oligos seems to moderately increase with increasing UV exposure. Crosslinking DNA to DNA with this method was found to yield 20 % more compared to crosslinking 2'-*O*-methylated RNA to DNA. This observation is in accordance with existing data and can be explained by

the increased dissociation constants of psoralen binding to RNA in comparison to DNA. Photo-crosslinking performed with free TMP showed the slowest rate, with however a steady increase with increasing radiant exposure, allowing for a 60 % maximal yield for the longest exposure time, in the same range as methods 1 and 3. Photo-crosslinking with free psoralen compounds takes place in three steps: intercalation of the molecule into the duplex, light-induced monoaddition to one strand and a following light-induced diaddition leading to the formation of an interstrand crosslink. Depending on the moiety of the psoralen molecule at which the cycloaddition takes place, two different monoadducts can be formed. While one of the monoadducts, involving the furan-side of psoralen, has the ability to form a diadduct upon addition of a second photon, thus leading to an interstrand crosslink, the pyrone-side monoadduct prevents the formation of a second one. Even though the development of psoralen derivatives like 4,5',8-trimethylpsoralen allowed to shift the balance of monoadducts towards the formation of interstrand crosslinks and additionally improved the overall crosslinking rate, our data shows a significantly reduced photo-crosslinking rate for free psoralen compared to the other methods. This decreased crosslinking yield may, in addition, be due to the low solubility of psoralen in water, or its dissociation from the nucleic acid duplex and the resulting low intercalation rate, or a combination thereof. Using modified and directly crosslinkable oligonucleotides, as in methods 1 and 3, bypasses the limitations associated with the proper intercalation of the photosensitive compound within the duplex, possible unselective crosslinking, low solubility or the formation of inactive monoadducts and therefore ensures high crosslinking rates together with mostly constant crosslinking yields.

We also studied the influence of temperature on the photo-induced crosslinking on chips. To allow for temperature control, we assembled a stand-alone UV source encased in aluminum housing suitable for cooling to lower temperatures. The same model Nichia 356 nm LED was used as in the biochip exposure system. We selected four characteristic radiant exposure values (1 J/cm^2, 5 J/cm^2, 20 J/cm^2 and 80 J/cm^2) for which crosslinking was performed either at room temperature or at 4 °C. The resulting gradients are shown in Supplementary Figure 4.

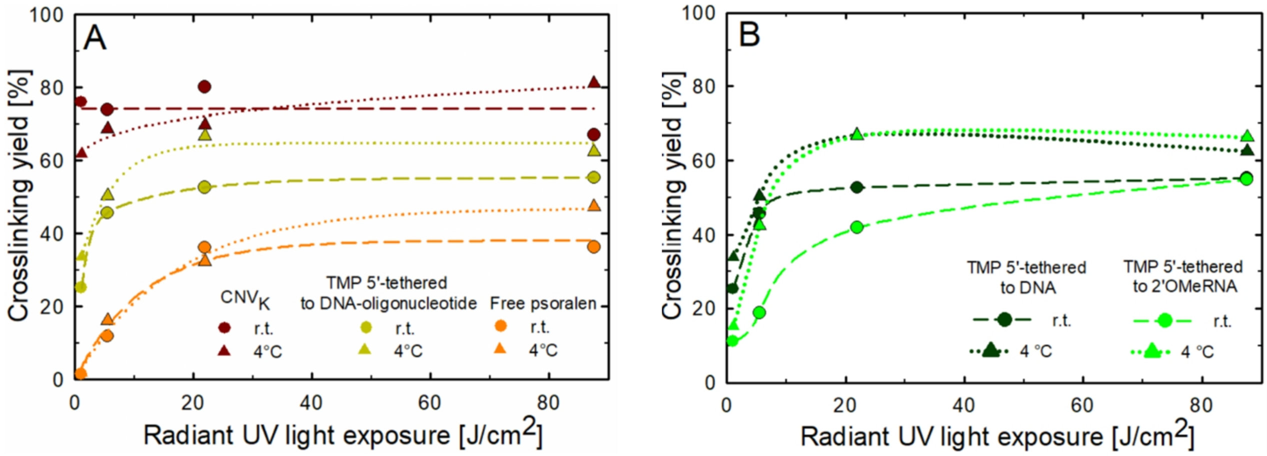

Supplementary Figure 4. Radiant UV light exposure gradients for different crosslinking methods. A stand-alone high-power UV LED served as light source providing 365 nm UV light at an intensity of 80 mW/cm^2. All data points were recorded on individual biochips. **A** Crosslinking was performed at room temperature (*dashed line*) and at 4 °C (*dotted line*) using either ^{CNV}K modified oligonucleotides (*dark red*), free TMP (*orange*) or TMP modified DNA oligonucleotides (yellow). **B** Crosslinking was performed at room temperature (*dashed line*) and at 4 °C (*dotted line*) using either TMP modified DNA (*light green*) or TMP modified 2'-*O*MeRNA oligonucleotides (*dark green*).

Slight differences in the crosslinking yield recorded at room temperature (Supplementary Figure 3 versus Supplementary Figure 4) can likely be attributed to differences in experimental variability. For all crosslinking experiments performed at reduced temperature, we observed a significant increase in maximal crosslinking efficiency, from 5 % for ^{CNV}K and up to 15 % for free psoralen compared to room temperature crosslinking, an effect that has previously been noticed for psoralen compounds. This effect is even more pronounced for the crosslinking of methylated RNA oligonucleotides, with an increase in yield of up to 30 % when performed at 4 °C, now in the same range as the crosslinking yield between DNA strands. In addition, our data shows that the maximum crosslinking yield at 4 °C is achieved at a similar radiant exposure for ^{CNV}K and psoralen-modified oligonucleotides. However, the crosslinking rate for free psoralen appears slower at 4 °C than at room temperature.

Repeated cycles of crosslinking. We wished to determine the maximum achievable crosslinking efficiency by iterating through cycles of hybridization and photo-crosslinking. This approach seems particularly important for free psoralen-mediated crosslinking, since it has been observed before that free psoralen is incapable of saturating DNA-binding sites in equilibrated solutions, due to its low solubility in water. To do so, biochips were fabricated as previously described and hybridized to their Cy3-labeled complementary oligonucleotides. Photocrosslinking was then

performed at 4 °C and at a radiant exposure of 25 J/cm^2 (Supplementary Figure 2 versus Supplementary Figure 3). After exposure, the arrays were thoroughly washed and underwent the next cycle of hybridization/crosslinking, for a total of 6 cycles. Results are shown in Supplementary Figure 5.

Repeating cycles of crosslinking (CL) leads to different results based on the CL method: while the CL yield slowly increases for ^{CNV}K and psoralen-modified oligos (Supplementary Figure 5. *Top*.), in case of method 2 (free psoralen), constant and relatively stable crosslinking yields of around 60 % were measured. Of particular note are the decreasing fluorescence intensities observed for crosslinking with free psoralen over multiple CL cycles compared to the increasing fluorescence intensities for the other methods (Supplementary Figure 5. *Bottom*). Although TMP favors the generation of furan-side monoadduct, the low fluorescence intensities observed even after multiple crosslinking cycles might be due to the additional formation of pyrone-side monoadducts, which do not undergo a second [2+2] cycloaddition and are therefore inactive in the formation of interstrand crosslinks. Moreover, inactive monoadducts simultaneously block the crosslinking sites for further psoralen intercalations. The further decrease of fluorescence intensity over several CL cycles can partially be explained with the degradation of the biochip surface due to repeated exposures to reagents, solvents and UV light. But since this effect is equivalent for all methods under investigation and is assumed to amount to a loss of around 10 % after the performance of 6 CL cycles, the contribution of another reason to this behavior is likely but would require further investigation.

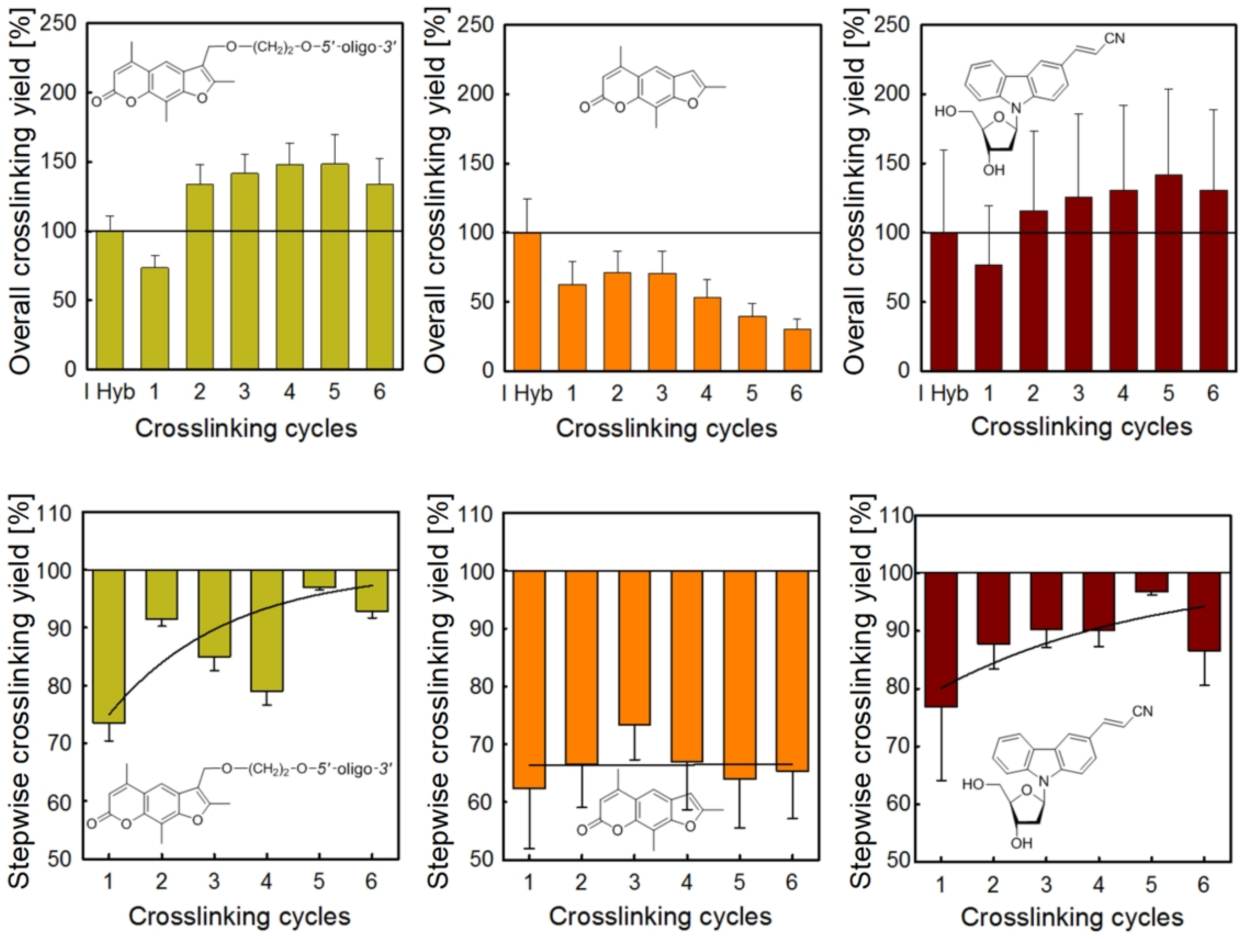

Supplementary Figure 5. Repeated cycles of crosslinking (CL), each consisting of a preliminary hybridization step to a Cy3-labeled complementary strand and a subsequent crosslinking event, followed by washing. Crosslinking was performed using 5'-psoralen modified oligonucleotides (*yellow*), free psoralen (*orange*) and ^{CNV}K-modified oligonucleotides (*dark red*) at 4 °C and a radiant exposure of 25 J/cm^2. **Top**. (Duplicate of Fig. 3d) Overall crosslinking yields [%] during repeated cycles of crosslinking. Crosslinking yield is determined as the change of fluorescence intensity after UV exposure and wash, and relative to the fluorescence intensity recorded for the initial hybridization. This hybridization was defined as 100 % CL efficiency. Error bars correspond to the standard deviation. **Bottom**. Stepwise crosslinking efficiencies [%] during repeated cycles of crosslinking. Crosslinking efficiency is determined as the change of fluorescence intensity after UV exposure and wash, and relative to the fluorescence intensity recorded after the corresponding hybridization step. Error bars correspond to the standard deviation.

Another important parameter to consider is the photobreakdown of free psoralen molecules. This phenomenon was observed for psoralens incapable of intercalating into the duplex, therefore remaining in solution. Following radiant exposure led to their photodestruction and therefore required fresh crosslinking compounds to be added in order to

achieve sufficiently high crosslinking yields. Since photobreakdown occurs for free psoralens not intercalated into duplexes and the fact that we perform thorough washing steps, leading to the complete removal of any remaining ingredients of the crosslinking solution, we assume that this aspect could at most only make a small contribution to the decreasing crosslinking efficiency. For crosslinking performed with modified oligonucleotides, a steady increase in fluorescence intensity was recorded, exceeding the values for the initial hybridization, although the crosslinking efficiency remained constant. This observation is unlikely to be a sign of unselective crosslinking occurring with increasing repetitions of crosslinking cycles as the hybridization temperature ensures perfect match hybridizations but is rather evidence of the fact that a single hybridization with crosslinkable oligonucleotides does not bind to all available surface-bound DNA templates. This hypothesis is supported by the fact that a saturation of the fluorescence intensity is finally reached after 5 to 6 hybridization and crosslinking cycles. Hybridizing with a large excess of Cy3-labeled complementary oligonucleotides could reduce the number of cycles required for a complete binding of all template strands on the biochip. A similar trend can be seen for the repeated CL cycles of psoralen-modified 2'-*O*MeRNA oligonucleotides. The corresponding CL efficiencies together with the fluorescence intensities are shown in Supplementary Figure 6.

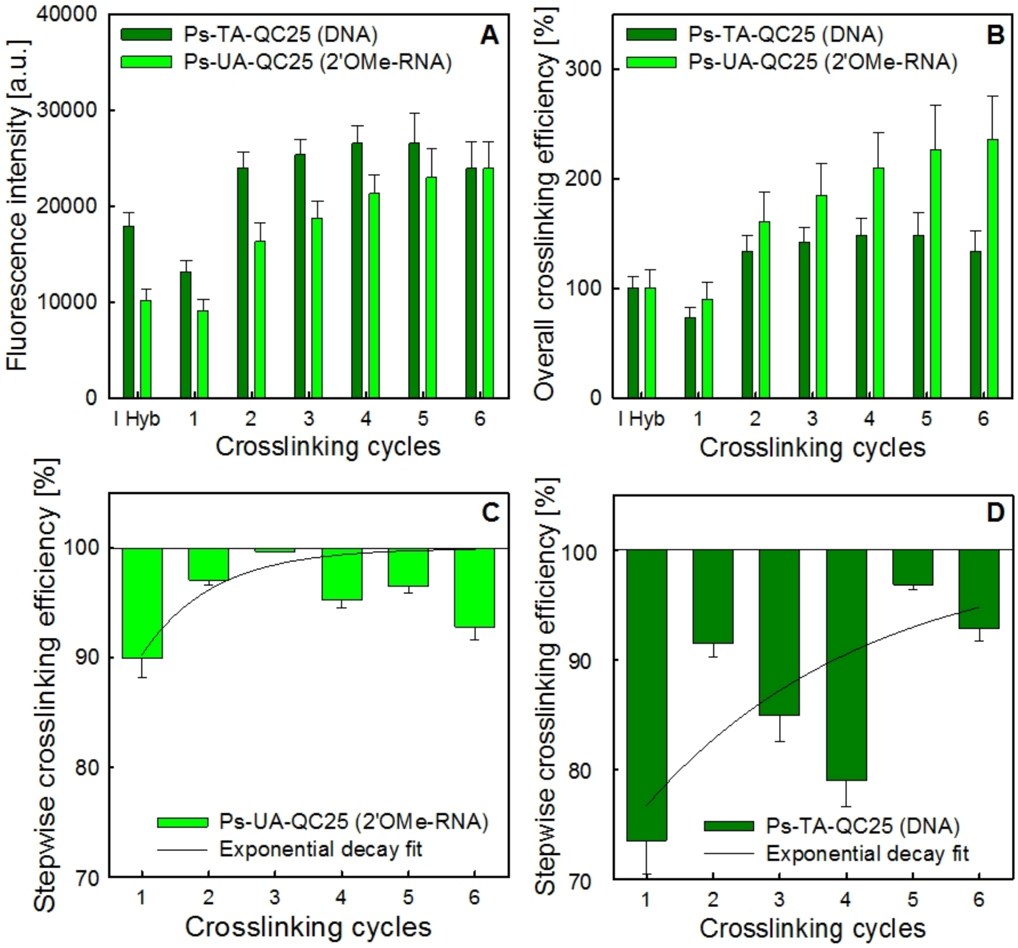

Supplementary Figure 6. Repeated cycles of crosslinking (CL) for 5'-psoralen (Ps) modified DNA oligonucleotides (*dark green*) and 5'-Ps modified 2'-*O*-MeRNA oligonucleotides (*light green*), each cycle consisting of a hybridization and a subsequent crosslinking step at 4 °C and a radiant exposure of 25 J/cm^2. Crosslinking efficiency is determined as the change of fluorescence intensity after UV exposure and wash. **A** Fluorescence intensities (arbitrary units) recorded during repeated cycles of crosslinking. Error bars correspond to the standard deviation. **B** Overall crosslinking efficiencies [%] over repeated cycles of crosslinking. Crosslinking efficiency is determined relative to the fluorescence intensity recorded for the initial hybridization step. This initial hybridization was defined as 100 % CL efficiency. Error bars correspond to the standard deviation. **C** and **D** Stepwise crosslinking efficiencies [%] over repeated cycles of crosslinking for psoralen modified 2'-*O*MeRNA (**C**) as well as DNA oligonucleotides (**D**). Crosslinking efficiency is determined relative to the fluorescence intensity recorded after each corresponding hybridization step. The initial hybridization was defined as 100 % CL efficiency. Error bars correspond to the standard deviation.

While absolute hybridization values for methylated RNA probes are initially lower than that of their DNA equivalents, repeating cycles of hybridization/crosslinking eventually yield similar hybridization intensities for DNA:DNA and DNA:2'-*O*MeRNA duplexes. However, in terms of CL efficiency, maximum CL with the methylated RNA oligonucleotide

required fewer cycles than for the corresponding DNA oligonucleotide and interestingly, although crosslinking of RNA:DNA duplexes is known to be less effective compared to double-stranded DNA, equally high efficiencies have been obtained.

Standard deviations for repeated cycles of crosslinking were determined by error propagation:

$$\Delta x = CL_{eff.} * \sqrt{\left(\frac{\sigma(x_{CL})}{I(x_{CL})}\right)^2 + \left(\frac{\sigma(x_{Hyb})}{I(x_{Hyb})}\right)^2}$$

where $CL_{\mathrm{eff.}}$ represents the crosslinking efficiency, I the average fluorescent intensity for either the hybridization (x_{Hyb}) or the crosslinking (x_{CL}) of probe x and σ the corresponding standard deviation. For determining the error of overall crosslinking efficiencies, the error propagation was calculated based on the initial hybridization of the experiment. For stepwise crosslinking efficiencies, the fluorescent intensity and standard deviation of each corresponding hybridization step was taken into account.

Biochip hybridization for crosslinking. The biochips were hybridized to Cy3-labeled complementary DNA or 2'-*O*-methylated RNA oligonucleotides purchased from Eurogentec, as described by Sack et al. Depending on the crosslinking method, the complementary oligonucleotides were either unmodified or contained a single ^{CNV}K unit (cyanovinylcarbazole) or a 5'-psoralen (4,5',8-trimethylpsoralen) modification. Oligonucleotide sequences together with their modifications are listed in Supplementary Table 2.

Supplementary Table 2. Sequences of complementary oligonucleotide probes used for crosslinking.

#	Probe	Sequence (5' to 3')	Modification	Chemistry
1	TA_QC25-Cy3_DNA	TAG ACC AGG GTG GTT CAT GAT GAT GAC	3'-Cy3	DNA
2	psoralen-TA-QC25-Cy3_DNA	TAG ACC AGG GTG GTT CAT GAT GAT GAC	5'-psoralen<br>3'-Cy3	DNA
3	psoralen-UA-QC25-Cy3_2'-*O*MeRNA	UAG ACC AGG GUG GUU CAU GAU GAU GAC	5'- psoralen<br>3'-Cy3	2'-*O*-MeRNA
4	^{CNV}K-QC25-Cy3_DNA	GAC CAX GGT GGT TCA TGA TGA TGA C	X = ^{CNV}K<br>3'-Cy3	DNA

The deprotected biochips were hybridized in a self-adhesive hybridization chamber (Grace Biolabs SA200) with 300 µl of a hybridization mix consisting of 150 µL 2x MES hybridization buffer, 110 µL nuclease free water, 13.3 µL acetylated BSA (10 mg/ml) and 26.7 µL of the 100 nM labeled complementary oligonucleotide. After incubation for 30 minutes, or 2 hours for the 2'-*O*MeRNA oligonucleotide, in a hybridization oven (Boekel Scientific) at 42 °C, the biochips were washed in non-stringent wash buffer NSWB (SSPE; 0.9 M NaCl, 0.06 M phosphate, 6 mM EDTA, 0.01 % Tween20) for 2 minutes, in stringent wash buffer SWB (100 mM MES, 0.1 M NaCl, 0.01 % Tween20) for 1 minute, followed by a short wash in final wash buffer FWB (0.1x SSC) for a few seconds, after which they were dried in a microarray centrifuge.

Crosslinking exposures. In order to examine the efficiency of photoinduced crosslinking on the chip surfaces, crosslinking experiments were performed *via* three different methods, using either psoralen 5'-tethered to oligonucleotides (method 1), free psoralen (method 2) or ^{CNV}K-modified oligonucleotides (method 3). A self-adhesive hybridization chamber was attached to the biochip surface and filled with 1x MES hybridization buffer or, in the case of method 2, with a 12.48 µM solution of psoralen in 1x MES (adding 1 µL of a 1 mg/mL psoralen stock solution in ethanol). Crosslinking was performed by exposing the biochip to 365 nm UV light at an output intensity of 80 mW/cm^2 for different time periods. In so doing, crosslinking efficiency can be measured as a function of specific radiant

exposure, in J/cm^2. The output of UV light intensity was adjusted using a calibrated intensity meter (SÜSS Micro-Tec 1000). After crosslinking, the biochips were first washed for 6 minutes in nuclease free water, followed by the same washing procedure used for the initial hybridization (2 min NSWB, 1 min SWB, 10 s FWB) and dried using a microarray centrifuge.

In preliminary experiments, crosslinking with the psoralen-modified oligonucleotide was also performed under dry conditions; the crosslinkable oligonucleotide was hybridized to its complementary DNA substrate. After washing off any remaining uncrosslinked oligonucleotides, the array was dried in a microarray centrifuge. Photo-induced crosslinking was performed by exposing the dry biochip to UV light according to the settings mentioned above. Since the resulting fluorescent intensities indicated a roughly 50 % decreased efficiency for crosslinking under dry conditions, all further crosslinking reactions were performed in solution, specifically 1 × MES hybridization buffer.

UV light exposure gradients. UV light exposure gradients were performed for each crosslinking method and for different oligonucleotide chemistries. To do so, biochips were synthesized with a 5mer dT-linker first and then the single sequence 5'-GTC ATC ATC ATG AAC CAC CCT GGT CTA. In order to accommodate various radiant exposures on the same biochip surface, the synthesis area was divided in twelve rectangular areas of identical size, alternating exposed and unexposed areas. Each exposed area received a specific amount of UV light (increasing from 1 to ~100 J/cm^2). The unexposed areas, or gaps, served to prevent unintended photo-crosslinking due to scattering of light. The biochips were washed and dried as described above.

To investigate the influence of temperature on the crosslinking efficiency, specific radiant exposure values (1, 5, 20 and 80 J/cm^2) were recorded again using a stand-alone high-power 365 nm UV LED (Nichia NVSU333A U365 surface-mount LED). For each radiant exposure, the photoinduced crosslinking was performed at room temperature and at 4 °C. Due to the nature of the stand-alone UV LED, each biochip was used for a single specific radiant exposure only. After crosslinking, the biochips were washed and dried as described above.

Supplementary Table 3. Sequence and crosslinking exposures used in Figure 3. Red and blue sequences are given in Supplementary Table 1.

Probe name	Color level	Crosslinking exposure (J/cm^2)	Modification	Chemistry
psoralen-TA-QC25-Cy3_DNA	G0	0	3'-Cy3<br>5'-psoralen C2	DNA
	G36	11		
	G72	22		
	G109	33		
	G145	44		
	G182	55		
	G218	66		
	G255	88		

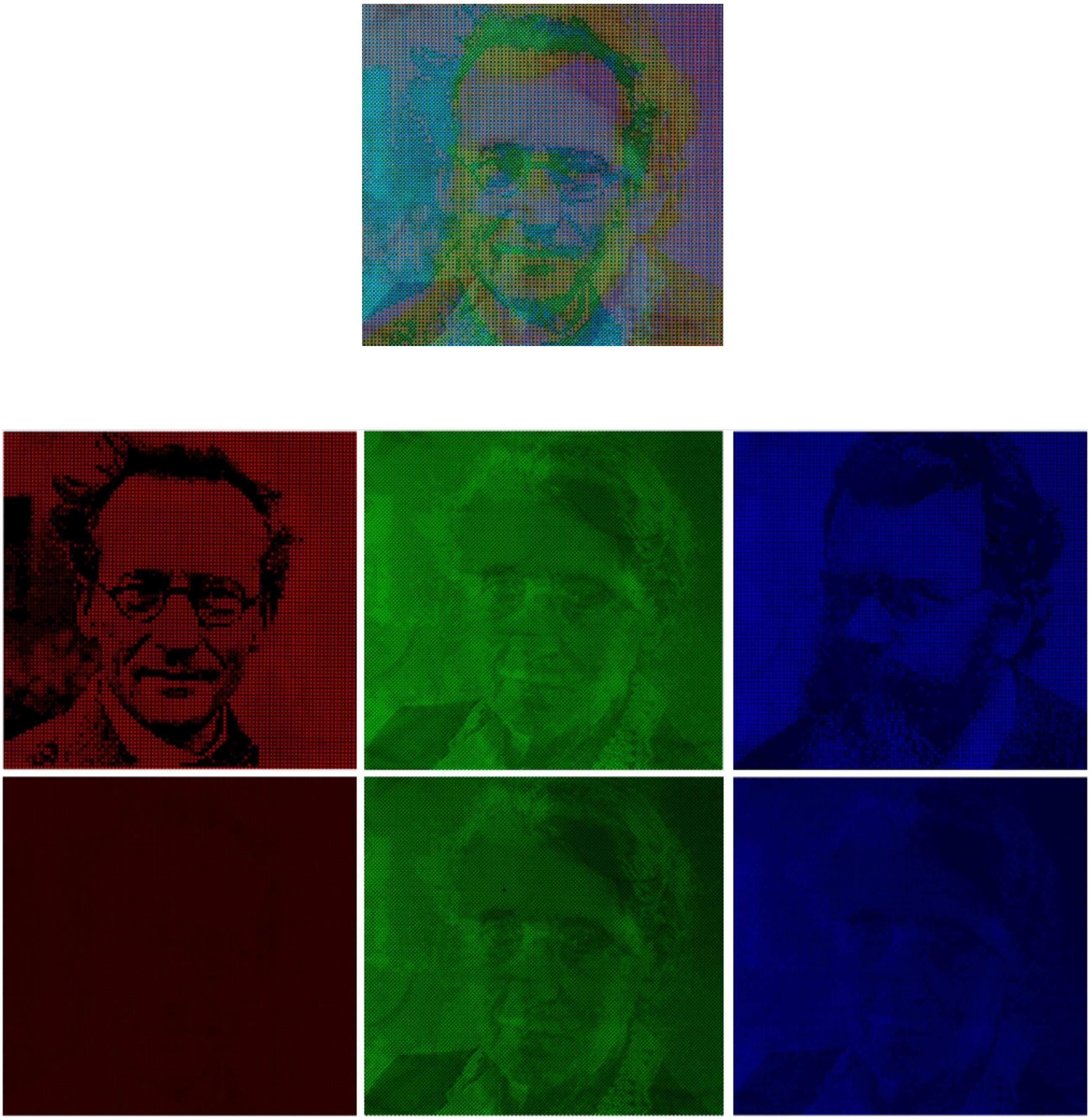

Supplementary Figure 7. Crosslinking steganography image combining images of Erwin Schrödinger (red channel), Lise Meitner (green channel) and Ludwig Boltzmann (blue channel). The steganograph consists of stacked images of the three physicists (top). Instead of encoding the full color information in each pixel, each color was segregated to separate pixels according to the pattern of a Bayer filter. The top image is a three color fluorescent scan after hybridization and crosslinking. The separated color channels of this scan reveal the component portraits (middle). The identity of the hidden image is revealed by washing the surface with water. After washing, only the crosslinked image of Lise Meitner is retained (bottom). Partial excitation of Cy3 fluorescence by the blue (488 nm) laser results in a weak image of Lise Meitner in the blue channel after wash-off. Original photographs © Vienna University Archive.

RNase A processing. Known effective substrates for RNase A (from bovine pancreas; Sigma-Aldrich R6513) were synthesized on a biochip (as described above) along with control strands with deoxythymidine replacing uridine. The substrates were based on those described by Kelemen et al. modified for surface-based cleavage and detection as shown in Table S4. RNase A cleavage takes place at the phosphate linkage on the 3' of the RNA nucleotide.

Supplementary Table 4. Biochip substrates for RNase A.

**RNase substrates**	**Non-cleavable controls**
Surface - T_{15}-dA-rU-Cy3	Surface - T_{15}-$(dA)_2$-dT-dA-Cy3
Surface - T_{15}-$(dA)_2$-rU-dA-Cy3	Surface - T_{15}-$(dA)_3$-dT-$(dA)_2$-Cy3
Surface - T_{15}-$(dA)_3$-rU-$(dA)_2$-Cy3	Surface - T_{15}-$(dA)_4$-dT-$(dA)_3$-Cy3
Surface - T_{15}-$(dA)_4$-rU-$(dA)_3$-Cy3	Surface - T_{15}-$(dA)_2$-dT-dA-Cy3

Cleavage efficiency was determined by immersing the chip into a 50 mL Falcon tube containing 40 mL of 100 nM RNase A in 0.1 MES buffer. After the desired cleavage time (Fig. 4a), the cleavage reaction was halted by quickly washing the surface in water and drying in an argon steam. A fluorescence scan was used to quantify loss of Cy3 fluorescent intensity of the substrates relative to the control. For the subsequent time points the chip was submitted to another cycle of cleavage and quantification. Efficiency is very similar for the last two substrates, AArUAAA and AAAruAAAA, and somewhat slower for the shorter substrates.

Uracil-DNA glycosylase processing. Enzymatic cleavage of DNA strands by means of dU incorporation and subsequent exposure to uracil-DNA glycosylase is a commonly used approach to enzymatic cleavage. Nevertheless, little is known about the sequence context-dependent efficiency of this process, particularly on surfaces. To evaluate this enzymatic cleavage approach, we synthesized DNA biochips also with dU by means of 5'-NPPOC dU phosphoramidites supplied by ChemGenes. The biochips were designed to include multiple substrates for uracil-DNA glycosylase, particularly one to nine dU nucleotides randomly embedded in a DNA context. The single or multiple dU-containing sequences were synthesized (using the maskless array synthesis approach described above) on a deoxythymidine 15mer linker to the glass surface. Synthesis continued on the 5' end, after the last dU, with a mixed base DNA 25mer (GAC CAG GGT GGT TCA TGA TGA TGA C). After synthesis and deprotection, the biochips were first hybridized with a Cy3-labeled complement to this 25mer, followed by incubation with a 0.015 U/µl UDG in 1x UDG Reaction Buffer for one hour

(New England Biolabs). After the cleavage step, the biochips were washed to remove UDG and rehybridized with the labeled 25mer. Cleavage was quantified by loss of fluorescence intensity relative to the initial hybridization. UDG-based cleavage is relatively inefficient, and multiple dU incorporations and incubation times of several hours were necessary to approach 80 % cleavage.

TURBO DNase and lambda exonuclease processing. Enzymes such as DNase I, its highly efficient engineered version TURBO DNase, and lambda exonuclease have proven to be of value for the degradation of DNA strands on biochip surfaces. While DNase I and especially TURBO DNase are highly efficient enzymes that degrade DNA non-specifically, lambda exonuclease catalyzes the removal of 5' mononucleotides from nucleic acids. In order to perform such enzymatic processing on biochip surfaces, the linker must be of sufficient length to prevent enzymes as e.g. lambda exonuclease from being stalled by the glass surface. In order to investigate DNase processing on solid surfaces, biochips were designed consisting of a dT-linker with varying length (T_1 to T_{20}) and a 25mer sequence serving as hybridization target. The locations of all sequences were randomized across the array surface and the oligonucleotides were synthesized according to the method described above. After synthesis, the chips were first hybridized to the 5'-Cy3 labeled 25mer complement (QC25) and subsequently incubated with either TURBO DNase (1U, 37 °C, 30 min) or lambda exonuclease (1U, 37 °C, 5 hours). The previous hybridization was then repeated to monitor the DNA degradation. The corresponding fluorescence intensities (indicated in arbitrary units) are plotted over the linker length and shown in Supplementary Figure 8.

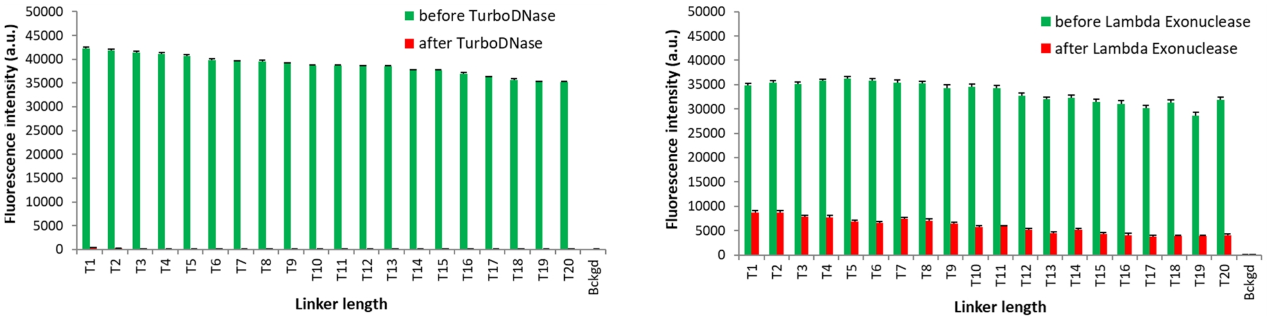

Supplementary Figure 8. DNase processing on surfaces. DNA strands with varying dT-linker length (T_1 to T_{20}) and a 25mer target sequence for hybridizations were synthesized on the chip surfaces, hybridized to their 5'-Cy3 labeled complement and incubated with either TURBO DNase (**left**) or lambda exonuclease (**right**). Subsequently, the arrays were rehybridized. Fluorescence intensities are shown in arbitrary units. Error bars indicate the SEM.

While a short incubation with TURBO DNase led to the complete degradation of the DNA strands, the degradation with lambda exonuclease required a significantly longer incubation time, due to the presence of 5'-OH instead of 5'-PO_4 ends, and a long linker length (>20mer).

In a subsequent experiment, a 3'-Cy3 labeled, 5'-psoralen modified 15mer oligonucleotide (primer) was hybridized to the surface-tethered DNA template and subsequently photo-crosslinked according to the method described above. The DNA template strands with varying dT-linker length (T_1 to T_5, T_{10}, T_{15} and T_{20}) were then enzymatically degraded using either TURBO DNase or lambda exonuclease. The corresponding fluorescence intensities (indicated in arbitrary units) that were recorded after the photo-induced crosslinking and after the enzymatic treatment are plotted over the linker length and shown in Supplementary Figure 9.

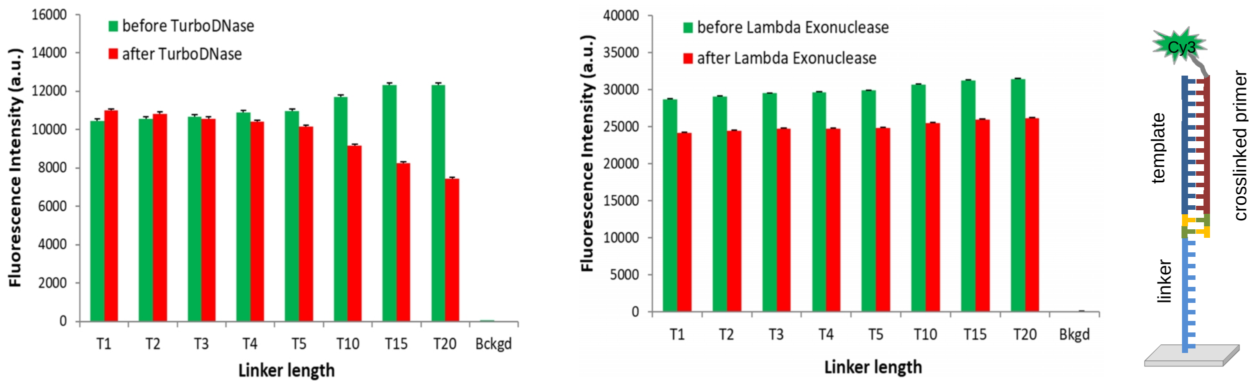

Supplementary Figure 9. DNase processing on biochip surfaces. DNA strands with varying dT-linker length (T_1 to T_5, T_{10}, T_{15} and T_{20}) and a random 15mer sequence (primer complement) were synthesized on the array surfaces. The strands were then hybridized to their 3'-Cy3, 5'-Psoralen modified 2'-*O*MeRNA complementary oligonucleotide (primer) and subsequently photo-crosslinked. The arrays were incubated with either TURBO DNase (**left**) or lambda exonuclease (**right**). The data bars represent the fluorescence intensities after the photo-induced crosslinking (*green*) and the remaining intensities obtained after the enzymatic treatment (*red*). Fluorescence intensities are shown in arbitrary units. Error bars indicate the SEM.

In contrast to lambda exonuclease which processes in the 5' to 3' direction and removes 5' mononucleotides from duplex DNA, TURBO DNase degrades dsDNA nonspecifically. In order to ensure that only the DNA template strands are enzymatically degraded, 2'-*O*-methylated RNA oligonucleotides were used as photo-crosslinkable primers. Our data indicate that lambda exonuclease is stalled at crosslinks which is in accordance to existing literature. As an endonuclease, TURBO DNase can bypass crosslink positions on DNA substrates and cleave crosslinked primers from the template strand and hence, from the surface. However, such cleavage can be prevented by choosing an appropriate linker length that is long enough to ensure a specific distance from the glass surface, but at the same time short enough to hinder TURBO DNase from cleaving between the crosslink and the surface.

Enzymatic on-chip DNA primer elongation. The on-chip enzymatic polymerization of a branched or crosslinked primer generates double-stranded DNA, or, with the use of appropriate polymerases and triphosphates, a second strand of another nucleic acid, with both strands covalently bound to the surface. In some applications, the template strand can then be degraded enzymatically. We primarily used 5'-psoralen-modified oligonucleotides to covalently attach DNA primers to single-stranded DNA templates on the surface. This approach is synthetically simpler and faster than the branched approach, but both are similarly effective. After template crosslinking, the addition of polymerase, NTPs and extension buffer allowed for the primer to be extended. Two sets of templates were designed, each consisting of the reverse complement of the primer on the 3' end, after the linker to the surface, and an additional 30mer serving as actual transcription template. In addition, templates presenting mismatching bases to the primer complement were synthesized. Control sequences were designed to either be the sequence of the elongated primer (or “template complement”, positive control) or the transcription template itself (negative control). Each biochip was divided into 4 identical subarrays, three being used for hybridization and crosslinking with 5'-psoralen primers, while the remaining subarray served as a hybridization and crosslinking control with 5'-psoralen, 3'-Cy3-labeled probes to verify successful crosslinking. After 4 crosslinking cycles, the primer extension was initiated by the addition of the polymerase (DNA Polymerase I, large (Klenow) fragment). Subsequently, competitive strand displacement hybridization was conducted, wherein either the enzymatically-extended DNA strands (PrEx_2) or their corresponding DNA templates (PrEx_1) were hybridized to their 5'-Cy3 labeled complements (Supplementary Figure 10). DNA sequences are given in Table S5. In so doing, the recorded fluorescence intensity indicates the presence of the extended primer, which in turn verifies both the crosslinking and polymerization steps. As a final test, the DNA template was enzymatically partly degraded using lambda exonuclease and the same hybridization was repeated. The corresponding results are shown in Supplementary Figure 11.

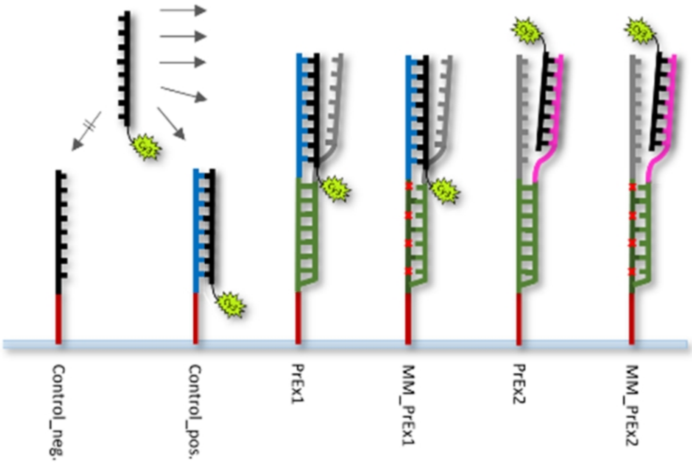

Supplementary Figure 10. Hybridization scheme for the enzymatic on-chip DNA primer elongation. Fluorescence intensities result from the hybridization of a 5'-Cy3 labeled 30mer oligonucleotide (GLD1) to the nucleic acid strands on biochip. Six different sequences are synthesized onto the surface: “Control_neg.” is to remain single-stranded throughout the hybridization experiments and serves as the negative control. “Control_pos.” functions as positive control that hybridizes to the 5'-Cy3 labeled 30mer oligonucleotide. For the sequences “PrEx1” and “MM_PrEx1” the oligonucleotide probe hybridizes in a competitive strand displacement manner to the surface-bound DNA elongation template. For “PrEx2” and “MM_PrEx2” the labeled probe hybridizes to the enzymatically elongated primer, again in a competitive strand displacement manner. The sequences “MM_PrEx1” and “MM_PrEx2” contain 4 mismatches in the primer complement.

Due to the design of the template strands and the fact that the outcome of the primer extension is a covalently-bound dsDNA, the labeled probes in the final hybridization step compete for binding with either the template (binding to the extended primer, PrEx2 and MM_PrEx2) or with the extended primers themselves (binding to the templates, PrEx1 and MM_PrEx1).

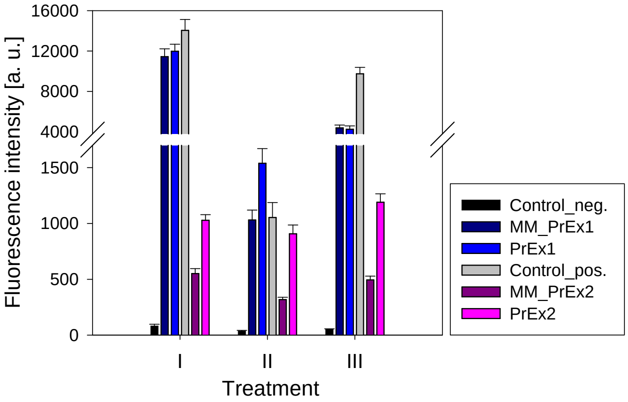

Supplementary Figure 11. Enzymatic on-chip primer elongation. Fluorescence intensities are shown in arbitrary units and result from the hybridization of a 5'-Cy3 labeled 30mer oligonucleotide to the sequences on the biochip surface. The labeled oligonucleotide hybridizes either to the ssDNA template on the array surface or the enzymatically elongated primer in a competitive strand displacement manner. Treatment (I) indicates the initial strand displacement hybridization after primer extension, (II) the following enzymatic degradation of the surface-bound DNA template and (III) a repetition of the initial hybridization. Error bars indicate the standard deviation.

As shown in Supplementary Figure 11 the fluorescence intensities corresponding to the binding to sequences PrEx1 and their mismatches-including equivalents MM_PrEx1 were found to be weaker than that of the positive control, where the hybridization assay is non-competitive. Although the difference seems to be quite small, the signal is based on a competitive strand displacement and is therefore assumed to be marginal. Simultaneously, a hybridization signal was recorded for PrEx2 sequences, indicating the presence of elongated primers. In addition, the difference in fluorescence intensity between PrEx2 and MM_PrEx2 suggests that mismatches in the primer region significantly affect the CL efficiency due to poor hybridization and result in low extension rates. Next, the DNA template strand of the duplex was enzymatically degraded. The complete cleavage of the sequences from surface is highly inefficient since the crosslink obstructs further enzymatic processing towards the surface in the case of lambda exonuclease (see 3.c above). Finally, the biochip was again hybridized to the Cy3-labeled probe. We found a clear decrease in the signal intensity of the Cy3 probes hybridized to surface-bound strands, PrEx1, MM_PrEx1 and the positive control, illustrating significant degradation of the templates. This decrease, 3-fold for PrEx1 and MM_PrEx1, was more modest for the positive control due to the short length of this sequence, bringing it closer to the biochip surface and which likely affects enzymatic processing. Conversely, the fluorescence intensity corresponding to the Cy3-probe: PrEx2 duplex increased, indicating stronger binding, which is consistent with a degradation of the template and the resulting

absence of competition for binding to the extended primer. For further template degradation the linker between the primer complement and the array surface needs to be elongated.

Supplementary Table 5. Sequences included in the biochip design for the primer extension experiments. All sequences are attached to the glass surface via a 15mer T-linker. Any unused space within the biochip synthesis area serves as background reference.

#	Sequence name	Sequence (5' to 3')	Description
1	Control_neg	GAT GAT GTA TGG CAC ATG ATT CTA TGG TAA	Negative control; remains single stranded throughout the whole experiment
2	Control_pos	TTA CCA TAG AAT CAT GTG CCA TAC ATC ATC	Positive control; reference for the strand displacement hybridization
3	PrEx_1	TTA CCA TAG AAT CAT GTG CCA TAC ATC ATC GTC ATC ATC ATG AAC CAC CCT GGT CTA	PrimerExtension_1: Long DNA strand that consists of a complementary primer sequence and an additional sequence as template for the primer extension. In the strand displacement hybridization, the extended DNA part competes with the added labeled oligonucleotide for hybridization to the template strand.
4	MM_PrEx_1	TTA CCA TAG AAT CAT GTG CCA TAC ATC ATC GTC ATG ATC ATC AAC GAC CGT GGT CTA	Mismatches_PrimerExtension_1: Similar to “PrEx_1” but contains 4 mismatches in the complementary primer sequence.
5	PrEx_2	GAT GAT GTA TGG CAC ATG ATT CTA TGG TAA GTC ATC ATC ATG AAC CAC CCT GGT CTA	PrimerExtension_2: Long DNA strand that consists of a complementary primer sequence and an additional sequence as template for the primer extension. In the strand displacement hybridization, the template DNA part competes with the added labeled oligonucleotide for hybridization to the extended DNA strand.
6	MM_PrEx_2	GAT GAT GTA TGG CAC ATG ATT CTA TGG TAA GTC ATG ATC ATC AAC GAC CGT GGT CTA	Mismatches_PrimerExtension_2: Similar to “PrEx_2” but contains 4 mismatches in the complementary primer sequence.

Supplementary References

1. C. Agbavwe, C. Kim, D. Hong, K. Heinrich, T. Wang, M.M. Somoza, Efficiency, error and yield in light-directed maskless synthesis of DNA microarrays, Journal of Nanobiotechnology 2011, 9. DOI: 10.1186/1477-3155-9-57.
2. A. Offner, Wavelength and coherence effects on the performance of real optical projection systems. Photographic Science and Engineering 1979, 23, 374.
3. S. Singh-Gasson, R. Green, Y. Yue, C. Nelson, F. Blattner, M. Sussman, F. Cerrina, Maskless fabrication of light-directed oligonucleotide microarrays using a digital micromirror array, Nature Biotechnology 1999, 17, 974 - 978. DOI: 10.1038/13664
4. M. Sack, N. Kretschy, B. Rohm, V. Somoza, M.M. Somoza, Simultaneous Light-Directed Synthesis of Mirror-Image Microarrays in a Photochemical Reaction Cell with Flare Suppression, Analytical Chemistry 2013, 85, 8513-8517. DOI: 10.1021/ac4024318.
5. N. Kretschy, A.-K. Holik, V. Somoza, K.-P. Stengele, and M. M. Somoza, Next Generation o-Nitrobenzyl Photolabile Groups for Light-Directed Chemistry and Microarray Synthesis Angewandte Chemie International Edition 2015 54(29), 8555–8559. DOI: 10.1002/anie.201502125.
6. M. Sack, N. Kretschy, K. Hölz, A.-K. Holik, V. Somoza, K.-P. Stengele, and M. M. Somoza, Express Photolithographic DNA Microarray Synthesis with Optimized Chemistry and High-Efficiency Photolabile Groups, Journal of Nanobiotechnology 2015 14(14). DOI: 10.1186/s12951-016-0166-0.
7. J. Lietard, K. Hölz, M. M. Somoza, A High-Power 365nm UV LED Mercury Arc Lamp Replacement for Photochemistry and Chemical Photolithography, ACS Sustainable Chemistry and Engineering 2016 5(1) 828–834. DOI: 10.1021/acssuschemeng.6b02175
8. K. Hölz, J. Hoi, E. Schaudy, V. Somoza, J. Lietard, M. M. Somoza, High-Efficiency Synthesis of Reverse (5′→3′) Oligonucleotides on Complex DNA Microarrays, Scientific Reports 2018, 8, 15099. DOI: 10.1038/s41598-018-33311-3
9. J. Lackey, D. Mitra, M. M. Somoza, F. Cerrina, M. Damha, Acetal Levulinyl Ester (ALE) Groups for 2'-Hydroxyl Protection of Ribonucleosides in the Synthesis of Oligoribonucleotides on Glass and Microarrays 2009 Journal of the American Chemical Society, 131(24), 8496. DOI: 10.1021/ja9002074.
10. J. Lietard, D. Ameur, M. Damha , M. M. Somoza, High-Density RNA Microarrays Synthesized in situ by Photolithography, Angewandte Chemie International Edition 2018, 57, 15257. DOI: 10.1002/anie.201806895

11. Pieles, U., and Englisch, U. (1989) *Psoralen covalently linked to oligodeoxyribonucleotides: synthesis, sequence specific recognition of DNA and photo-cross-linking to pyrimidine residues of DNA*, Nucleic Acids Research 17, 285-299.

12. Yoshimura, Y., and Fujimoto, K. (2008) *Ultrafast Reversible Photo-Cross-Linking Reaction: Toward in Situ DNA Manipulation*, Org. Lett. 10, 3227-3230.

13. Smith, S. I., and Brodbelt, J. S. (2010) *Rapid Characterization of Cross-links, Mono-adducts, and Non-covalent Binding of Psoralens to Deoxyoligonucleotides by LC-UV/ESI-MS and IRMPD Mass Spectrometry*, The Analyst 135, 943-952.

14. Fujimoto, K., Yamada, A., Yoshimura, Y., Tsukaguchi, T., and Sakamoto, T. (2013) *Details of the Ultrafast DNA Photo-Cross-Linking Reaction of 3-Cyanovinylcarbazole Nucleoside: Cis–Trans Isomeric Effect and the Application for SNP-Based Genotyping*, Journal of the American Chemical Society 135, 16161-16167.

15. Isaacs, S. T., Shen, C.-K. J., Hearst, J. E., and Rapoport, H. (1977) *Synthesis and characterization of new psoralen derivatives with superior photoreactivity with DNA and RNA*, Biochemistry 16, 1058-1064.

16. Cole, R. S. (1971) *Psoralen monoadducts and interstrand cross-links in DNA*, Biochimica Biophysica Acta 254, 30-39.

17. Spielmann, H. P., Sastry, S. S., and Hearst, J. E. (1992) *Methods for the large-scale synthesis of psoralen furan-side monoadducts and diadducts*, Proceedings of the National Academy of Sciences of the United States of America 89, 4514-4518.

18. Yeung, A. T., Jones, B. K., and Chu, C. T. (1988) *Photoreactivities and thermal properties of psoralen cross-links*, Biochemistry 27, 3204-3210.

19. Ashwood-Smith, M. J., and Grant, E. (1974) *Effect of temperature on dose-dependent changes in sedimentation characteristics of bacterial DNA produced, in vivo, by near-ultraviolet irradiation and 8-methoxypsoralen*, Cryobiology 11, 160-169.

20. Smith, K. C., and Leary, M. E. (1967) *Photoinduced DNA-Protein Cross-Links and Bacterial Killing: A Correlation at Low Temperatures*, Science 155, 1024.

21. Sakamoto, T., Tanaka, Y., and Fujimoto, K. (2015) *DNA Photo-Cross-Linking Using 3-Cyanovinylcarbazole Modified Oligonucleotide with Threoninol Linker*, Organic Letters 17, 936-939.Kelemen BR, Klink TA, Behike MA, Eubanks SR, Leland PA, Raines RT., *Hypersensitive substrate for ribonucleases*, Nucleic Acids Research **1999** 27(18):3696-701.

22. M F Berger and M L Bulyk *Universal protein-binding microarrays for the comprehensive characterization of the DNA-binding specificities of transcription factors* Nature Protocols **2009** 4 (3) 393-411, 10.1038/nprot.2008.195
23. J Wang, T Li, Y Bai, Y Zhu and Z Lu *Fabrication of unimolecular double-stranded DNA microarrays on solid surfaces for probing DNA-protein/drug interactions* Molecules **2003** 8, 153-168, 10.3390/80100153
24. G Rossetti, P D Dans, I Gomez-Pinto, I Ivani, C Gonzalez and M Orozco *The structural impact of DNA mismatches* Nucleic Acids Research **2015** 43 (8) 4309-21, 10.1093/nar/gkv254
25. D Suck *DNA Recognition by DNase I* Journal of Molecular Recognition **1994** 7, 65-70, 10.1002/jmr.300070203
26. J E Herrera, J B Chaires *Characterization of preferred deoxyribonuclease I cleavage sites* Journal of Molecular Biology **1994** 236 (2), 405-11, 10.1006/jmbi.1994.1152
27. P G Mitsis and J G Kwagh *Characterization of the interaction of lambda exonuclease with the ends of DNA* Nucleic Acids Research **1999** 27 (15) 3057-3063, 10.1093/nar/27.15.3057
28. T T Perkins, R V Dalal, P G Mitsis and S M Block *Sequence-Dependent Pausing of Single Lambda Exonuclease Molecules* Science **2003** 301 (5641) 1914-1918, 10.1126/science.1088047
29. W B Mattes *Lesion selectivity in blockage of lambda exonuclease by DNA damage* Nucleic Acids Research **1990** 18 (13) 3723-3730, 10.1093/nar/18.13.3723
30. A Tsourkas, M A Behlke and G Bao *Hybridization of 2'O-methyl and 2'-deoxy molecular beacons to RNA and DNA targets* Nucleic Acids Research **2002** 30 (23) 5168-5174, 10.1093/nar/gkf635
31. G Zhao and Y Guan *Polymerization behavior of Klenow fragment and Taq DNA polymerase in short primer extension reactions* Acta Biochimica et Biophysica Sinica **2010** 42 (10) 722-728, 10.1093/abbs/gmq082
32. N Schormann, R Ricciardi and D Chattopadhyay *Uracil-DNA glycosylases – Structural and functional perspectives on an essential family of DNA repair enzymes* Protein Science **2014** 23, 1667-1685, 10.1002/pro.2554
33. S S Parikh, C D Mol, G Slupphaugh, S Bharati, H E Krokan and J A Tainer *Base excision repair initiation revealed by crystal structures and binding kinetics of human uracil-DNA glycosylase with DNA* The EMBO Journal **1998** 17 (17) 5214-5226, 10.1093/emboj/17.17.5214
34. B Xia, Y Liu, W Li, A R Brice, B N Dominy and W Cao *Specificity and catalytic mechanism in family 5 uracil DNA glycosylase* Journal of Biological Chemistry **2014** 289 (26) 18413-18426, 10.1074/jbc.M114.567354
